# Effect of dietary protein on plasma insulin-like growth factor-1, growth, and body composition in healthy term infants: a randomised, double-blind, controlled trial (Early Protein and Obesity in Childhood (EPOCH) study)

**DOI:** 10.1017/S0007114515004456

**Published:** 2015-11-20

**Authors:** Guy Putet, Jean-Marc Labaune, Katherine Mace, Philippe Steenhout, Dominik Grathwohl, Veronique Raverot, Yves Morel, Jean-Charles Picaud

**Affiliations:** 1 Service de Neonatologie, Hopital de la Croix-Rousse, Hospices Civils de Lyon, F-69004 Lyon, France; 2 Universite Claude Bernard Lyon1, F-69100 Villeurbanne, France; 3 Nestec, 55 Avenue Nestlé, Vevey, Switzerland; 4 Laboratoire d’hormonologie et Endocrinologie Moléculaire, Centre de Biologie et Pathologie Est, Hospices Civils de Lyon, 69677 Bron, France; 5 Rhone-Alpes Human Nutrition Research Center, Hopital Lyon Sud, F-69310 Pierre-Benite, France

**Keywords:** Infants, Dietary proteins, Obesity, Growth, Fat mass

## Abstract

The effect of protein intake on growth velocity in infancy may be mediated by insulin-like growth factor-1 (IGF-1). This study aimed to determine the effects of formulae containing 1·8 (F1·8) or 2·7 g (F2·7) protein/418·4 kJ (100 kcal) on IGF-1 concentrations and growth. Healthy term infants were randomly assigned to receive F1·8 (*n* 74) or F2·7 (*n* 80) exclusively for the first 4 months of life. A group of breast-fed infants (*n* 84) was followed-up simultaneously (reference). Growth and body composition were measured at 0·5, 4, 6, 12, 36, 48 and 60 months of life. The IGF-1 concentrations at 4 months (primary outcome) were similar in the F1·8 (67·1 (sd 20·8) ng/l; *n* 70) and F2·7 (71·2 (sd 27·5) ng/l; *n* 73) groups (*P*=0·52). Both formula groups had higher IGF-1 concentrations than the breast-fed group at 4 and 9 months of age (*P*≤0·0001). During the first 60 months of life, anthropometric parameters in the F1·8 group were lower compared with the F2·7 group, and the differences were significant for head circumference from 2 to 60 months, body weight at 4 and 6 months and length at 9, 12 and 36 months of age. There were no significant differences in body composition between these two groups at any age. We conclude that, in formula-fed infants, although increased protein intake did not affect the IGF-1 concentration during the first 12 months of life, it did affect length and head circumference growth, suggesting that factors other than IGF-1 could play roles in determining growth velocity.

The prevalence of obesity has now reached epidemic proportions, and prevention rather than treatment holds the best promise for curbing this phenomenon. Recent studies have shown that rapid weight gain during infancy is closely associated with subsequent risks of overweight and obesity^(^ ^1^ ^–^ ^5^ ^)^. Thus, early growth may be a key target for intervention.

The factors leading to rapid weight gain during infancy are not well understood. Studies comparing growth rates (primarily weight gain) in infants fed formula or complementary foods and those fed breast milk have suggested a positive association between high protein intake and rapid growth rate during infancy^(^ ^6^ ^,^ ^7^ ^)^. These observations are consistent with several studies showing higher growth rates (weight and length gains) in infants fed standard-protein formulae (2·3–2·5 g protein/418·4 kJ (100 kcal)) compared with those fed low-protein formulae (1·8–1·9 g protein/418·4 kJ (100 kcal))^(^ ^8^ ^,^ ^9^ ^)^, where the protein concentrations of the low-protein formulae were closer to that of mature human milk (about 1·5 g protein/418·4 kJ (100 kcal)). Furthermore, a recent large randomised trial showed that infants fed high-protein infant formula (2·9 g protein/418·4 kJ (100 kcal)) and follow-on formula (4·4 g protein/418·4 kJ (100 kcal)) during their 1st year of life had significantly higher weight and weight-for-length *Z*-scores (relative to the WHO standards) compared with infants fed low-protein infant formula (1·8 g protein/418·4 kJ (100 kcal)) and follow-on formula (2·2 g protein/418·4 kJ (100 kcal))^(^ ^10^ ^)^. In contrast, an earlier study showed no differences in weight, length or head circumference between infants fed standard-protein (2·7 g protein/418·4 kJ (100 kcal)) or low-protein formulae (1·9 g protein/418·4 kJ (100 kcal)) during the 1st year of life^(^ ^11^ ^)^. Part of the inconsistency in the growth data from different studies may reflect differences in the formula protein concentrations, the period during which protein intake was evaluated (early or late infancy or childhood) and/or the timing of growth measurements. Furthermore, growth data are difficult to compare between studies, as they are often presented in absolute values instead of *Z*-scores adjusted for age and sex.

The effect of protein intake during infancy on obesity later in life is even more equivocal. Both the presence^(^ ^12^ ^–^ ^14^ ^)^ and absence^(^ ^15^ ^,^ ^16^ ^)^ of a relationship between high protein intake during infancy and early childhood and later obesity development have been reported. However, these conclusions were based on observational studies that primarily measured protein intake during late infancy and early childhood – that is, during 9−24 months of age – and randomised trials are still lacking.

The mechanisms by which growth patterns in infancy may affect obesity risks later in life are not well established. The observation that formula-fed infants generally tend to have greater body mass and a higher risk of being obese later in life than breast-fed infants has given rise to the ‘early protein hypothesis’. According to this hypothesis, high protein intake early in life causes enhanced growth by stimulating insulin and insulin-like growth factor-1 (IGF-1) production^(^ ^17^ ^,^ ^18^ ^)^. In addition to promoting growth, increases in these hormones have been reported to stimulate adipogenic activity and adipocyte differentiation^(^ ^18^ ^)^, thereby increasing susceptibility to overweight and obesity at a later age. However, to date, a few studies have evaluated the effects of formulae that differ only in their protein content on IGF-1 concentration in infants.

In the Early Protein and Obesity in Childhood (EPOCH) study, our primary objective was to compare the IGF-1 concentrations at 4 months of age in infants exclusively fed low-protein (1·8 g protein/418·4 kJ (100 kcal)) or standard-protein (2·7 g protein/418·4 kJ (100 kcal)) formulae or breast milk (reference group) from birth up to 4 months of age. We hypothesised that infants fed formula with protein content close to that of breast milk would have IGF-1 concentrations similar to those of breast-fed infants and lower than those of infants fed a standard-protein formula. The secondary objectives were to determine the plasma or serum hormone (insulin, C-peptide) and glucose concentrations as well as to assess growth and body composition during the first 60 months of life in all groups of infants.

## Methods

### Study design

The EPOCH study was a randomised, double-blind, parallel-group, controlled, single-centre study comparing two groups of infants fed formula with a low or high protein content for the 1st year of life. An observational group of breast-fed infants was included from the Hôpital de la Croix-Rousse in Lyon, France, during the same period. The study was performed in accordance with the Helsinki Declaration and current good clinical practices guidelines. Both parents provided written informed consent before their infants were enrolled in the study. The study was approved by the ethics committee of Lyon (Comité Consultatif de Protection des Personnes dans la Recherche Biomédicale Lyon A).

### Study participants

Infants were recruited from the study site from mothers who had chosen to exclusively feed their infants formula or breast milk from birth until 4 months of age. The inclusion criteria included being full term (gestational age of 37−42 weeks), healthy, <7 d of age at the time of enrolment, between 2500 and 4200 g at birth, singleton birth and being born to a mother with a normal BMI (20−25 kg/m^2^) at the start of pregnancy and without a history of type 1 or type 2 diabetes. The exclusion criteria were having a mother with the following characteristics: gestational diabetes, smoking >5 cigarettes/d, drug dependency or infectious disease during pregnancy. Infants whose mothers/caregivers were not expected to comply with the study requirements or infants who were already participating in a different clinical study were also excluded.

### Study formulae

The study formulae were isoenergetic (285 kJ/100 ml (68 kcal/100 ml)), predominantly whey-based (70 % whey, 30 % casein) and contained sufficient quantities of carbohydrates, proteins, fats, vitamins and minerals to support the normal growth of term infants. The two study formulae had identical nutritional composition, except for protein and lactose concentrations: the low-protein formula (F1·8) contained 1·8 g protein/418·4 kJ (100 kcal), and the standard-protein formula (F2·7) contained 2·7 g protein/418·4 kJ (100 kcal). These protein concentrations were within the range of the European legal standards for infant formulae (1·8–3·0 g/418·4 kJ (100 kcal)) and for follow-on formulae (1·8–3·5 g/418·4 kJ (100 kcal))^(^ ^19^ ^)^. The F1·8 and F2·7 also corresponded to the lower and upper limits, respectively, of protein content in the follow-on formulae available in France. The lactose content in the formulae was adjusted to keep the same energy density in both formulae (F1·8: 11·2 g lactose/418·4 kJ (100 kcal) and F2·7: 10·4 g lactose/418·4 kJ (100 kcal)). The study sponsor manufactured, coded and blinded the formulae using four-letter codes. Neither the investigator and support staff nor the infants’ mothers/caregivers were aware of the identity of the formulae. Unblinding occurred after the statistical analyses of the primary and secondary outcomes were completed.

### Study procedure

The infants were stratified by sex and socio-cultural status (mother’s education level and ethnicity) and randomly allocated to the two formula-fed groups. Randomisation was performed using computer-generated randomisation sequences (SAS Institute) using a block design with block sizes of 8. Allocation concealment was performed by placing randomisation numbers in sealed, sequentially numbered envelopes.

The formula-fed infants were randomly assigned to receive F1·8 or F2·7. The demographic characteristics of all infants and their mothers (in both the formula-fed and breast-fed groups) and the following baseline characteristics of infants were recorded upon enrolment: sex; gestational age; delivery mode; weight, length and head circumference at birth; and medical history. The mothers’ BMI before pregnancy, weight gain and smoking status during pregnancy and general health status were also recorded.

The infants were exclusively fed their assigned formulae for 4 months starting at enrolment (i.e. at <7 d of age). Infants were excluded from the study if they were fed a formula other than the one they were assigned for >7 consecutive d during the first 4 months of life. They could start complementary feeding at 4 months but they had to maintain intake of their assigned formula (F1·8 or F2·7) as their follow-on formula until 12 months of age. Breast-fed infants had to be exclusively breast-fed for 3 months of age. At the mothers’ discretion, the breast-fed infants could be fed up to 200 ml/d of F1·8 during the 4th month. They could start complementary feeding at 4 months of age, but were instructed to use the F1·8 formula as a follow-on formula, if needed, until 12 months of age.

For the first 4 months, the parents/caregivers recorded the volume of daily formula intake of the formula-fed infants. For the breast-fed infants, any daily supplementary formula (F1·8) intake between 3 and 4 months was recorded. After 4 months, the volume of formula and amount of solid food intake of all infants were recorded during the 3 d before each visit and the compliance with the study formulae was assessed and recorded at each visit. The protein and energy intakes were estimated using a computer programme (Genesis R&D software; Esha Research) by a nutritionist.

Visits to the study centre took place at the age of 2 weeks (±2 d) and at 2, 4, 6, 9, 12, 36, 48 and 60 months (±1 week). At each visit, the investigator recorded the anthropometric measurements, reviewed the records of formula intake and evaluated the occurrence of adverse events (AE) and intake of any concomitant medications. Body composition was measured at 2 weeks and at 4, 6, 12, 36 and 60 months of age. Blood samples were drawn at 2 weeks, 4 months and 9 months of age. Blood hormone concentrations were measured at all these time points.

At 2 weeks and 4 months of age, the infants were fed at the study site for 10−15 min, and blood samples were collected 45 min after the start of feeding. If there was a delay of ≥60 min after starting feeding, blood samples were not collected. At 9 months of age, blood sampling was performed approximately 2 h after the last meal. The exact time of feeding and blood collection were recorded for each infant and used to ensure similar timing of feeding and blood sample collections across visits and infants. The infants’ weights before and after feeding were also recorded, in order to ascertain that a substantial amount of milk was ingested before measuring postprandial insulin concentrations.

### Outcome measures

The primary outcome measurement was the plasma IGF-1 concentration at 4 months, as it was the end of exclusive feeding with the study products, with weaning being allowed after 4 months of age. Secondary outcome measurements included the IGF-1 concentrations at 2 weeks and 9 months of age; postprandial insulin, C-peptide, IGF-binding protein (IGFBP)-2, IGFBP-3 and glucose concentrations at 2 weeks, 4 months and 9 months of age; weight, length and head circumference measurements at 2 weeks and 4, 6, 9, 12, 36, 48 and 60 months of age; and body composition measurements at 2 weeks and 4, 6, 12, 36 and 60 months of age.

For blood glucose concentrations, the analysis was performed on-site and immediately after sampling using standard laboratory methods. For IGF-1 analysis, 1 ml of blood was collected in EDTA-containing tubes and centrifuged at 4°C, and plasma aliquots were stored at −80°C until further analysis. For measurement of insulin, C-peptide, IGFBP-2 and IGFBP-3 concentrations, 1 ml of blood was collected in dry tubes and centrifuged at 4°C, and the serum was stored at −80°C. The IGF-1 and insulin concentrations were measured by immunoassay (IGF-1-RIACT and BI-INS-IRMA, respectively; Cis Bio International). Immunoradiometric assays were used to determine the concentrations of IGFBP-2 (DSL-7100; Diagnostic Systems Laboratories Inc.), IGFBP-3 (IM1992; Immunotech) and C-peptide (IM3639; Immunotech). The within/between assay precisions were, respectively, <3·8/<8 % for insulin, <3·1/<5·2 % for C-peptide, <3·4/<8·2 % for IGF-1, <5·6/<8·2 % for IGFBP-2 and <8·5/<7·4 % for IGFBP-3.

The infants’ weight, length and head circumference were measured as described previously^(^ ^20^ ^)^. The body composition of infants who were 6-months old or younger was measured by air-displacement plethysmography using the PEA POD system (Life Measurements Inc.). At 12, 36 and 60 months of age, the infants’ body composition was measured by dual energy X-ray absorptiometry (DEXA, Hologic 4500; Stephanix). Both fat and lean mass measurements were recorded. The quality of the DEXA scans was assessed as previously recommended^(^ ^21^ ^)^. The skinfold thickness (triceps, biceps, sub-scapular and abdominal) and mid-arm circumference were measured at 36, 48 and 60 months of age.

### Adverse events

AE were defined as illnesses or signs or symptoms (including abnormal laboratory findings) occurring or worsening during the course of the study. A serious AE (SAE) was any AE that was fatal or life-threatening, caused permanent harm, required or extended in-patient treatment at a hospital or that was considered medically relevant by the physician. The occurrence of AE and SAE was based on interviews with the parents or caregivers. The investigator assessed all AE for seriousness, causality and relation to the study formulae. All AE, whether or not they required medical intervention, were recorded and coded using the WHO Adverse Reaction Terminology version 07.4.

### Sample size

The primary outcome was the IGF-1 concentration at 4 months of age. Sample size calculation was based on detecting a 26 % difference in IGF-1 concentrations between the two formula-fed groups. A previous study showed an sd of 37·8 μg/l for IGF-1 concentrations at 4 months of age in infants who were fed F2·7 and F1·8^(^ ^22^ ^)^. At a two-sided significance level of 5 % and a power of 90 %, sixty-four infants per group were required to detect a difference. Assuming a 20 % rate of dropouts and missing data, seventy-seven infants had to be included in each group to attain the required power. The dropout rate in the breast-fed group was expected to be higher than that in the formula-fed groups due to reduced compliance to exclusive breast-feeding for 3 months. Assuming a 30 % dropout/non-compliance rate, eighty-four infants thus had to be enrolled in the breast-fed group.

### Statistical methods

Data from all randomised infants who had any intake of the study formulae and all breast-fed infants in the reference group were included in the intention-to-treat (ITT) analysis. The per-protocol (PP) analysis excluded data from infants with major protocol deviations or who had missing primary outcome data. In some instances, measurements could not be made in all infants, and the ITT numbers are therefore lower than those at inclusion.

In order to investigate whether socio-economic factors may influence the loss to follow-up, we performed a time-to-event analysis. The event of interest was the last visit seen before follow-up loss, with infants seen at 5 years considered as censored. We calculated the time to the last visit seen (days) and performed Cox-proportional hazard analysis with the treatment group (breast-fed, F2·7, F1·8), ethnicity (northern European or not), education (as outlined in Table 1) and smoking during pregnancy (yes/no) as covariates.

**Table 1:** Demographics and baseline characteristics of infants fed low-protein or standard-protein formulae (F1·8 or F2·7) or breast milk, intention-to-treat (Mean values and standard deviations; numbers and percentages)

	F1·8 (*n* 74)		F2·7 (*n* 80)		Breast-fed (*n* 84)	
Characteristics	Mean	sd	Mean	sd	Mean	sd
Infants						
Gestational age (weeks)	39·6	1·2	39·5	1·1	39·4	1·0
Age at enrolment (d)	3·1	1·0	3·3	0·9	3·2	0·8
Boys						
*n*	41		42		42	
%	55·4		52·5		50	
Caesarean births						
*n*	9		12		9	
%	12·1		15		10·7	
Infants breast-fed before enrolment						
*n*	8		7		84	
%	10·8		8·7		100	
Weight at birth (g)	3410	410	3380	380	3380	330
Length at birth (cm)	50·2	1·9	50·1	1·6	50·1	1·55
Head circumference at birth (cm)	34·5	1·1	34·7	1·1	34·6	1·1
Mothers						
Height (cm)	164·9	6·1	164·4	5·2	165·8	5·6
Weight before pregnancy (kg)	58·3	5·8	57·8	6·9	58·6	6·7
Weight gain during pregnancy (kg)	13·2	4·0	13·1	4·2	12·4	4·0
BMI before pregnancy (kg/m^2^)	21·5	2·0	21·4	2·3	21·3	1·8
Smoking during pregnancy						
*n*	4		4		0	
%	5·4		5·0			
With chronic pathologies						
*n*	7		3		1	
%	9·4		3·7		1·1	
With other pathologies						
*n*	3		1		1	
%	4·0		1·2		1·1	
Northern or Western European in origin						
*n*	54		60		70	
%	72·9		75		83·3	
Professional diploma or lower						
*n*	13		15		6	
%	17·5		18·7		7·1	
Baccalaureate +<4 years of university						
*n*	30		31		19	
%	40·5		38·7		22·6	
≥4 years of university						
*n*	31		34		59	
%	41·8		42·5		70·2	

F1·8, low-protein formula (1·8 g/418·4 kJ (100 kcal)); F2·7, standard-protein formula (2·7 g/418·4 kJ (100 kcal)).

The primary outcome measurement was the plasma IGF-1 concentration at 4 months. A hierarchical test procedure on the comparison between F1·8 *v*. F2·7 and F1·8 and breast-feeding was applied. Thus, these two hypotheses were controlled for by multiple testing on a 5 % level. All other outcomes and comparisons were considered as secondary and were not controlled for by multiple testing. We followed the ICH E9 guideline, Statistical Principles for Clinical Trials and interpreted small *P* values on secondary outcomes as a ‘flagging device’, indicating interesting results.

All outcome measures were compared between the groups by ANCOVA using the type of formula intake as the explanatory variable and controlling for sex, socio-cultural status and ethnicity as covariates. The hormone and glucose concentrations were log transformed in order to achieve approximately normally distributed residuals; the results are presented as ratios with 95 % CI. In addition, the growth parameters were also compared with the World Health Organization^(^ ^23^ ^)^ child growth standards. All the statistical analyses were performed using SAS version 9.2 (SAS Institute).

## Results

### Study population

**Fig. 1:**
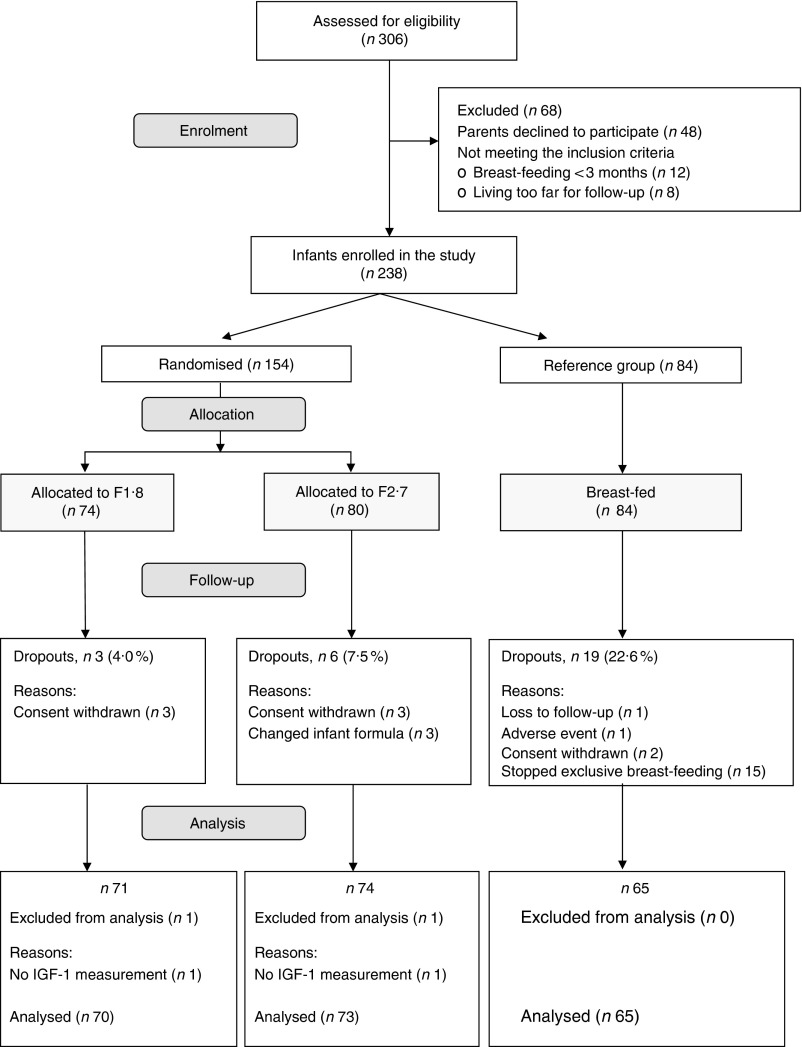
Infants’ participation throughout the randomised, double-blind study of infant formula. F1·8, low-protein formula (1·8 g/418·4 kJ (100 kcal)); F2·7, standard-protein formula (2·7 g/418·4 kJ (100 kcal)); IGF-1, insulin-like growth factor-1.

Between June 2006 and October 2007, 238 infants were enrolled in the EPOCH study and were included in the ITT analysis set (*n* 74 in the F1·8 group, *n* 80 in the F2·7 group and *n* 84 in the breast-fed group) (Fig. 1). Data from thirty infants were excluded from the ITT analysis, owing to dropout from the study or difficulty obtaining IGF-1 measurements at 4 months of age (Fig. 1); none of these infants had IGF-1 measurements. Thus, the results of the primary outcome from the PP analysis were similar to that of the ITT analysis, and only the ITT analyses are presented in this report.

The groups were comparable with respect to the infants’ and mothers’ anthropometric characteristics at baseline (Table 1). The socio-cultural status was different between the formula-fed and breast-fed groups, but not between the two formula-fed groups (Table 1). Eight mothers smoked during pregnancy, all of whom were in the formula-fed groups.

### Plasma hormone profile

The measurements of plasma IGF-1 concentrations, taken at 0·5 months and 4 and 9 months of age, were not significantly different between the formula-fed groups (Table 2). In the breast-fed group, the mean IGF-1 concentration was lower compared with the two formula-fed groups at 4 and 9 months of age. The IGFBP-3 concentrations were not different between the formula-fed groups at any time point. However, at 4 months of age, the IGFBP-3 concentrations were lower in the breast-fed than in the formula-fed groups, and these differences persisted at 9 months of age. The IGFBP-2 concentrations were significantly higher in the F1·8 than in the F2·7 group only at 4 months of age. In the breast-fed group, the IGFBP-2 concentration was higher compared with both formula-fed groups at 4 months, but only remained significantly higher compared with the F2·7 group at 9 months of age.

**Table 2:** Insulin-like growth factor-1 (IGF-l), IGF-binding protein (IGFBP)-2 and IGFBP-3 serum concentrations at 0·5 months, 4 and 9 months of age in infants fed the study formulae (F1·8 or F2·7) or breast milk, intention-to-treat‡ (Numbers; medians and interquartile ranges (IQR))

	F1·8			F2·7			Breast-fed		
Serum concentrations (ng/ml)	*n*	Median	IQR	*n*	Median	IQR	*n*	Median	IQR
0·5 months									
Total IGF-1	72	83·5	29·0	77	83·0	26·0	77	88·0	35·0
IGFBP-2	71	45·6	28·0	78	45·9	27·2	77	45·5	27·1
IGFBP-3	71	1·5	0·3	78	1·4	0·3	78	1·4	0·3
4 months									
Total IGF-1	70	66·0	24·0	73	63·0	42·0	65	46·0*†	23·0
IGFBP-2	70	28·7	15·3	73	22·9*	12·8	65	40·2*†	26·8
IGFBP-3	70	1·8	0·3	73	1·7	0·4	65	1·5*†	0·4
9 months									
Total IGF-1	61	62·0	37·0	69	63·5	33·5	57	43·0*†	24·0
IGFBP-2	61	24·9	12·9	69	24·2	10·8	57	27·0†	16·6
IGFBP-3	61	1·9	0·5	69	1·8	0·4	57	1·7*†	0·4

F1·8, low-protein formula (1·8 g/418·4 kJ (100 kcal)); F2·7, standard-protein formula (2·7 g/418·4 kJ (100 kcal)). * Significantly different from F1·8 (*P*<0·05).

† Significantly different from F2·7 (*P*<0·05).

‡ Inferential statistics by ANCOVA, correcting for sex, ethnicity and socio-cultural status.

The postprandial insulin and C-peptide concentrations were similar between the formula-fed groups at 0·5 months and 4 and 9 months of age, but were lower in the breast-fed infants at 2 weeks and 4 months of age (Table 3). There were no differences in glucose concentrations between the formula-fed groups at any time point, and the mean glucose concentration was slightly higher in the breast-fed group compared with the formula-fed groups only at 9 months of age.

**Table 3:** Insulin, C-peptide and glucose serum concentrations at 0·5 months, 4 and 9 months of age in infants fed the study formulae (F1·8 or F2·7) or breast milk, intention-to-treat‡ (Numbers; medians and interquartile ranges (IQR))

	F1·8			F2·7			Breast-fed		
Serum concentrations	*n*	Median	IQR	*n*	Median	IQR	*n*	Median	IQR
0·5 months									
Insulin (U/ml)	70	18·0	15·0	74	17·5	14·0	77	14·0*†	10·0
C-peptide (ng/ml)	70	2·1	1·3	73	2·3	1·3	77	1·4*†	1·1
Glucose (mmol/ml)	70	5·4	1·5	79	5·3	0·9	76	5·5	1·1
4 months									
Insulin (U/ml)	65	10·0	6·5	65	10·0	6·0	63	7·0*†	4·0
C-peptide (ng/ml)	65	2·2	1·1	65	2·1	1·4	63	1·2*†	1·1
Glucose (mmol/ml)	68	5·6	0·7	70	5·5	0·6	64	5·5	0·6
9 months									
Insulin (U/ml)	55	5·0	5·0	62	5·0	5·0	53	5·0	3·0
C-peptide (ng/ml)	55	2·3	1·7	62	2·1	1·0	53	2·0	1·2
Glucose (mmol/ml)	61	4·9	0·9	67	4·9	0·7	55	5·1†	0·6

F1·8, low-protein formula (1·8 g/418·4 kJ (100 kcal)); F2·7, standard-protein formula (2·7 g/418·4 kJ (100 kcal)). * Significantly different from F1·8 (*P*<0·05).

† Significantly different from F2·7 (*P*<0·05).

‡ Inferential statistics by ANCOVA, correcting for sex, ethnicity and socio-cultural status.

### Nutrient intake

**Fig. 2:**
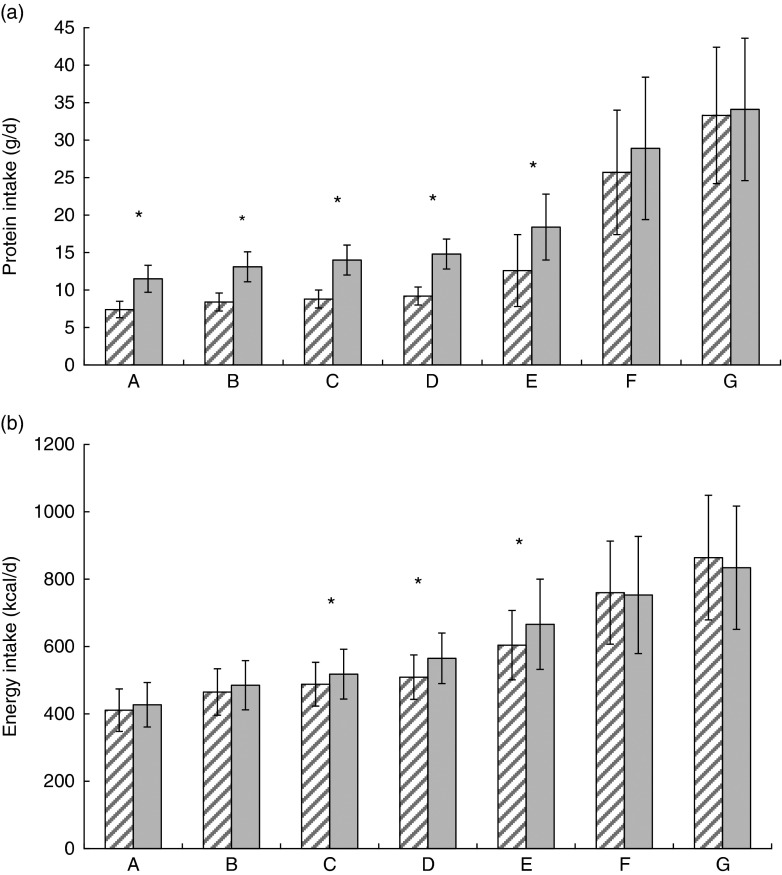
Daily protein (a) and energy (b) intakes between 0·5 and 1 month (A), 1 and 2 months (B), 2 and 3 months (C), 3 and 4 months (D) and at 6 months (E), 9 months (F) and 12 months of age (G) in infants fed low-protein (F1·8, 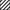) or standard-protein (F2·7, 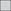) formula. Values are means, with standard deviations represented by vertical bars. * Significant difference between feeding groups (*P*<0·05). To convert kcal/d to kJ/d, multiply by 4·184.

The volume intakes are presented in Table 4. The mean protein intake was increased by 50–60 % in infants fed F2·7 when compared with F1·8 during the first 6 months of life (Fig. 2). There was also a slightly higher (+6−10 %) mean energy intake in the F2·7 group between 2 and 6 months of age (Fig. 2). This difference remained between 2 and 4 months of age only when the energy intake was expressed per kg body weight. After 6 months of age, and the introduction of complementary feeding, there were no differences in protein and energy intake. The breast-fed group was weaned at 170 (sd 43) d.

**Table 4:** Volume intakes (ml/d) of infants fed the study formulae (F1·8 or F2·7), intention-to-treat† (Numbers; mean values and standard deviations)

	F1·8			F2·7		
Infants age	*n*	Mean	sd	*n*	Mean	sd
0·5−1 month	70	613·1	93·7	75	637·0	98·2
1−2 months	70	694·3	103·1	75	724·3	109·3
2−3 months	71	728·1	96·5	71	772·8*	110·6
3−4 months	71	759·7	98·4	71	816·3*	112·4

F1·8, low-protein formula (1·8 g/418·4 kJ (100 kcal)); F2·7, standard-protein formula (2·7 g/418·4 kJ (100 kcal)). * Significantly different from F1·8 (*P*<0·05).

† Inferential statistics by ANCOVA, correcting for sex.

### Growth parameters

**Fig. 3:**
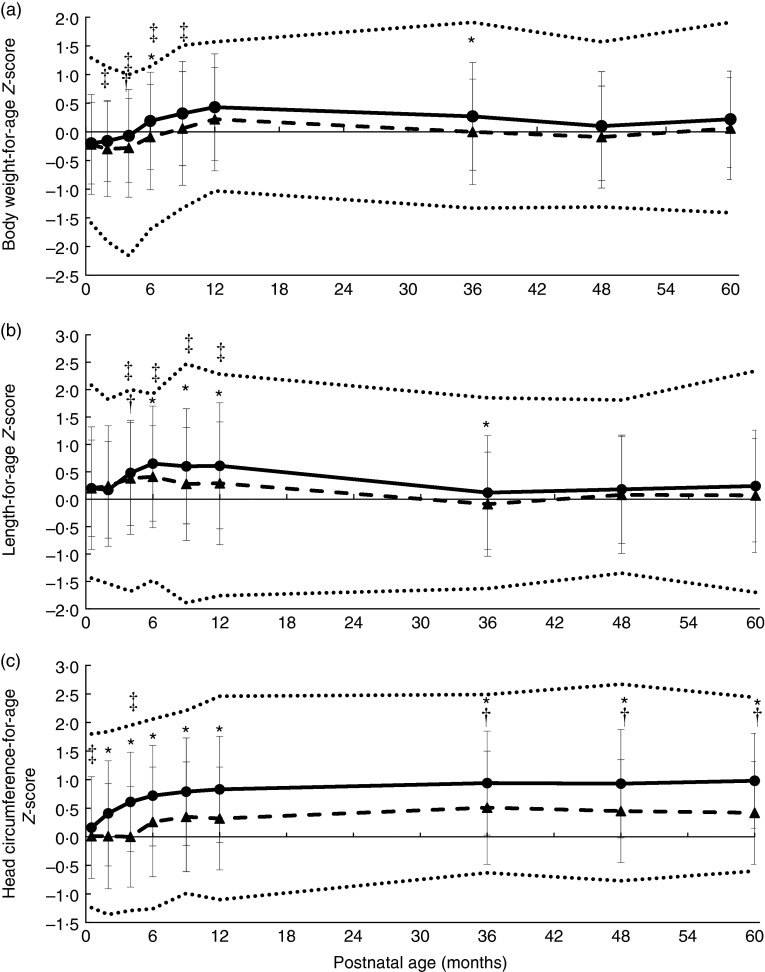
Weight-for-age (a), length-for-age (b) and head circumference-for-age (c) *Z*-scores during the first 60 months of life in infants fed a low-protein (F1·8, 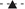) or standard-protein formula (F2·7, 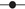). Values are means, with standard deviations represented by vertical bars. Upper and lower dotted lines indicate +2 sd and −2 sd of the reference group of breast-fed infants, respectively. * Significant difference between the two groups of formula-fed infants (*P*<0·05). † Significant difference between infants fed F1·8 and breast-fed infants (*P*<0·05). ‡ Significant difference between infants fed F2·7 and breast-fed infants (*P*<0·05).

There were no significant differences between the formula-fed groups in any of the growth measurements (weight, length, head circumference and BMI) at 0·5 months (Table 5). During the first 60 months of life, all anthropometric parameters in the F1·8 group were lower than those in the F2·7 group, and these differences were statistically significant for head circumference from 2 to 60 months, for body weight at 4 and 6 months and for length at 9, 12 and 36 months of age. There were significant differences in the *Z*-scores between the two formula-fed groups for body weight at 6 and 36 months, for length from 6 to 36 months and for head circumference from 2 to 60 months of age (Fig. 3).

**Table 5:** Anthropometric measurements between 0·5 and 60 months of age in infants fed the study formulae (F1·8 or F2·7) or breast milk, intention-to-treat‡ (Mean values and standard deviations; numbers)

	F1·8		F2·7		Breast-fed	
Anthropometric measurements	Mean	sd	Mean	sd	Mean	sd
0·5 months (*n*)	72		79		77	
Weight (kg)	3·61	0·45	3·61	0·39	3·64	0·4
Length (cm)	52·5	2·2	52·4	1·8	52·7	1·7
HC (cm)	35·7	1·2	35·8	1·1	36·0†	1·0
BMI (kg/m^2^)	13·1	0·9	13·1	1·0	13·1	1·0
2 months (*n*)	72		77		71	
Weight (kg)	5·23	0·56	5·32	0·59	5·16†	0·6
Length (cm)	58·5	2·3	58·3	2·1	58·2	1·8
HC (cm)	38·8	1·1	39·3*	1·3	39·1	1·1
BMI (kg/m^2^)	15·3	1·1	15·6	1·1	15·2†	1·3
4 months (*n*)	71		74		65	
Weight (kg)	6·54	0·7	6·70*	0·73	6·26*†	0·7
Length (cm)	63·8	2·3	64·1	2·3	63·2*†	2·4
HC (cm)	41·4	1·2	41·9*	1·3	41·4†	1·2
BMI (kg/m^2^)	16·0	1·4	16·3	1·3	15·7†	1·1
6 months (*n*)	71		72		63	
Weight (kg)	7·62	0·8	7·84*	0·87	7·41*†	0·7
Length (cm)	67·8	2·1	68·2	2·6	67·2*†	2·2
HC (cm)	43·2	1·3	43·7*	1·4	43·4	1·2
BMI (kg/m^2^)	16·6	1·4	16·8	1·3	16·4†	1·1
9 months (*n*)	68		69		62	
Weight (kg)	8·71	0·98	8·95	1·01	8·71	0·8
Length (cm)	71·8	2·4	72·5*	2·7	71·8†	2·8
HC (cm)	44·9	1·3	45·5*	1·5	45·2	1·3
BMI (kg/m^2^)	16·9	1·4	17·0	1·4	16·9	1·3
12 months (*n*)	67		69		60	
Weight (kg)	9·65	1·03	9·87	1·11	9·65	0·9
Length (cm)	75·8	2·8	76·6*	3·1	75·6†	2·8
HC (cm)	46·0	1·3	46·6*	1·5	46·4	1·3
BMI (kg/m^2^)	16·8	1·3	16·8	1·2	16·9	1·2
36 months (*n*)	63		66		58	
Weight (kg)	14·26	1·6	14·70	1·7	14·72	1·6
Length (cm)	95·4	3·5	96·0	4·0	96·0	3·5
HC (cm)	49·8	1·4	50·3*	1·5	50·4	1·3
BMI (kg/m^2^)	15·6	1·2	15·9	1·0	15·9	1·1
48 months (*n*)	61		62		57	
Weight (kg)	16·13	1·84	16·56	2·01	16·66	1·6
Length (cm)	103·3	4·2	103·8	4·2	104·3	3·5
HC (cm)	50·5	1·3	51·1*	1·5	51·2*	1·3
BMI (kg/m^2^)	15·1	1·1	15·3	1·0	15·3	1·1
60 months (*n*)	59		58		53	
Weight (kg)	18·62	2·22	19·10	2·20	18·98	2·0
Length (cm)	110·2	4·6	110·9	4·7	110·8	3·6
HC (cm)	51·0	1·4	51·8*	1·3	51·7*	1·2
BMI (kg/m^2^)	15·3	1·2	15·5	1·0	15·4	1·1

F1·8, low-protein formula (1·8 g/418·4 kJ (100 kcal)); F2·7, standard-protein formula (2·7 g/418·4 kJ (100 kcal)); HC, head circumference. * Significantly different from F1·8 (*P*<0·05).

† Significantly different from F2·7 (*P*<0·05).

‡ Inferential statistics by ANCOVA correcting for sex, ethnicity and socio-cultural status.

The comparisons of the anthropometric parameters and *Z*-scores between the formula-fed and breast-fed infants are presented in Table 5 and Fig. 3.

### Body composition

The fat mass and fat-free mass (percentage of body weight), as assessed by DEXA and the PEA POD system, were similar in the formula-fed groups during the first 60 months of life (Table 6). When compared with breast-fed infants, infants fed F1·8 exhibited a significantly higher fat mass and lower fat-free mass at 0·5 and 4 months of age. There were no differences in skinfold thickness and mid-arm circumference between the feeding groups (data not shown).

**Table 6:** Fat mass and fat-free mass between 0·5 and 60 months of age in infants fed the study formulae (F1·8 or F2·7) or breast milk, intention-to-treat† (Mean values and standard deviations; numbers)

	F1·8		F2·7		Breast-fed	
Measurements (%BW)	Mean	sd	Mean	sd	Mean	sd
0·5 months (*n*)‡	72		79		77	
Fat mass	13·2	3·4	12·7	3·4	11·8*	3·5
Fat-free mass	86·8	3·4	87·3	3·4	88·2*	3·5
4 months (*n*)‡	71		74		65	
Fat mass	26·1	3·9	24·9	4·2	24·2*	4·4
Fat-free mass	73·9	3·9	75·1	4·2	75·8*	4·4
6 months (*n*)‡	71		72		63	
Fat mass	26·2	4·5	25·9	4·2	25·4	4·7
Fat-free mass	73·8	4·8	74·1	4·2	74·6	4·7
12 months (*n*)§	56		59		50	
Fat mass	29·4	8·7	29·8	7·3	28·6	6·7
Fat-free mass	70·6	8·7	70·2	7·3	71·4	6·7
36 months (*n*)§	51		50		53	
Fat mass	28·3	5·1	28·2	3·9	28·3	4·4
Fat-free mass	71·7	5·1	71·8	3·9	71·7	4·4
60 months (*n*)§	51		50		53	
Fat mass	24·4	4·1	24·2	3·3	23·8	4·2
Fat-free mass	73·9	4·0	74·2	3·3	75·4	5·2

F1·8, low-protein formula (1·8 g/418·4 kJ (100 kcal)); F2·7, standard-protein formula (2·7 g/418·4 kJ (100 kcal)); BW, body weight. * Significantly different from F1·8 (*P*<0·05).

† Inferential statistics by ANCOVA correcting for baseline status and sex.

‡ Assessed by PEA POD.

§ Assessed by dual energy X-ray absorptiometry.

### Adverse events

A total of 753 AE were reported in 206 infants during the study. More infants in the F2·7 group (*n* 76, 95·0 %) had an AE compared with infants in the F1·8 (*n* 66, 89·1 %) or breast-fed (*n* 64, 76·1 %) groups. In all, eleven AE were rated as ‘probably’ or ‘certainly’ related to the study product. These included one case of eczema with respect to F2·7, one case of dyspepsia with respect to F1·8 and 1, 2, 1, 1 and 4 cases of eczema, allergy, rash erythematous, abdominal pain and weight decrease, respectively, related to human milk consumption.

In total, twenty-three SAE were reported in twenty-two infants (Table 7). Only one SAE (urticaria) in the breast-fed group was considered as formula related. One infant in the breast-fed group was withdrawn due to an SAE (fever) 10 d after birth, and three others dropped out during the follow-up period (between 6 and 12 months).

**Table 7:** Infants with serious adverse events, intention-to-treat (Numbers and percentages)

	F1·8 (*n* 74)		F2·7 (*n* 80)		Breast-fed (*n* 84)	
Serious adverse events*	*n*	%	*n*	%	*n*	%
Acute bronchiolitis	1	1·4	3	3·8	0	
Hypospadias	1	1·4	0		0	
Gastroenteritis	2	2·8	0		1	1·2
Fever	3	4·2	2	2·5	2	2·4
Pyelonephritis	1	1·4	0		1	1·2
Vomiting	1	1·4	0		0	
Feeding problems	1	1·4	0		0	
Coughing	1	1·4	0		0	
Urticaria	0		0		1	1·2†
Rhinitis	0		0		1	1·2
Respiratory condition	1	1·4	0		0	
Total	12		5		6	

F1·8, low-protein formula (1·8 g/418·4 kJ (100 kcal)); F2·7, standard-protein formula (2·7 g/418·4 kJ (100 kcal)).

* One infant had two serious adverse events.

† Considered related to the study formula.

## Discussion

In a population of healthy term infants, we did not observe any impact of protein intake on the plasma hormone profile (IGF-1, insulin and C-peptide concentrations) during the first 12 months of life. In infants fed the low-protein formula, growth during the first 60 months of life was lower than that in infants fed the standard-protein formula; however, the mean values for body weight, length and head circumference were within the normal WHO ranges for growth in both groups of formula-fed infants. As expected, we observed a significantly lower protein intake in infants fed the F1·8 *v*. F2·7 formula. Interestingly, although the two study formulae were isoenergetic, we also observed an unexpected higher energy intake during the first 6 months of life in the F2·7 group than in the F1·8 group. Such an increase in energy intake may be related to a higher overall volume of formula intake, as previously reported by Fleddermann *et al*.^(^ ^24^ ^)^ in infants fed a standard-protein formula (2·2 g/418·4 kJ (100 kcal)) when compared with a low-protein formula (1·89 g/418·4 kJ (100 kcal)). They reported that, although the volume intake per meal was not different, the higher intake in the high-protein group could be explained by a higher meal frequency as a result of lower satiety. Although dietary proteins have been shown to be the most satiating macronutrient in adults, little is known on their impact on satiety in infants, and this warrants further exploration. Nevertheless, it should be noted that the observed difference in energy intake in the present study was lower when adjusted to per kg body weight.

Except for one study published recently^(^ ^25^ ^)^, the majority of studies that have examined the correlation between protein intake and IGF-1 concentrations in infants were performed comparing formula-fed and either breast-fed infants or infants fed cow milk^(^ ^26^ ^–^ ^30^ ^)^. Thus, linking any differences in IGF-1 concentrations in infants fed these diets solely to their protein concentrations may be difficult. On the other hand, in the EPOCH study, the formulae differed in protein concentration only. Our results revealed no significant differences in plasma IGF-1 concentrations between infants exclusively fed F1·8 or F2·7 formula. This is in contrast with the positive associations between IGF-1 concentration and both energy and protein intakes in infants reported previously^(^ ^28^ ^,^ ^31^ ^)^. Socha *et al*., in a similarly designed study, reported a significantly higher mean IGF-1 concentration at 6 months of age in infants fed a high-protein formula (2·9 g/418·4 kJ (100 kcal)) than in infants fed a low-protein formula (1·77 g/418·4 kJ (100 kcal)) during the first 4 months of age^(^ ^27^ ^)^. In that study, as in the EPOCH study, both formula-fed groups had higher IGF-1 concentrations than the exclusively breast-fed infants after 4 months.

Several differences in study designs may explain the discrepancies between the EPOCH and previous studies – for example, in the Childhood Obesity Project Study Group (CHOP) study^(^ ^10^ ^)^, the enrolment of infants was performed later than that in the EPOCH study (median, 14 *v*. 3 d), and some infants received breast milk for a few weeks, which may have had a significant metabolic impact. The significantly higher postprandial insulin and C-peptide concentrations in formula-fed infants than in breast-fed infants at 0·5 months of age observed in our study and others^(^ ^32^ ^)^ highlight the importance of early randomisation. Further, in the CHOP study, the formulae used were casein-predominant, whereas in the EPOCH study whey-predominant formulae were used. Interestingly, casein, but not whey protein, has been previously shown to promote IGF-1 secretion^(^ ^33^ ^)^, and this may have impacted the results. In addition, in the CHOP study, there was a huge increase in the protein content of the formula used after 4 months of life^(^ ^10^ ^,^ ^25^ ^)^. Indeed, the follow-on formulae started at 4 months of age contained 2·2 and 4·4 g protein/418·4 kJ (100 kcal), respectively. Therefore, this resulted in a sharp increase in protein intake in the high-protein group, reaching a protein:energy ratio close to whole cows’ milk (5·5 g/418·4 kJ (100 kcal)), as underlined by Michaelsen & Greer^(^ ^34^ ^)^. On the other hand, in the EPOCH study, the infants consumed the same formula (either F1·8 or F2·7) as a follow-on formula up until 12 months of age, and the breast-fed infants all received F1·8, if necessary. Thus, assuming that formula feeding between 4 and 6 months is still predominant, it can be speculated that the daily protein intake during this period was probably much higher in infants fed the high-protein formula in the CHOP study than in infants fed either the F1·8 or the F2·7 formula in our study. Finally, in the CHOP study, blood sampling for hormonal status assessment was performed 2 months after the introduction of the follow-on formula and beginning of complementary food. In the EPOCH study, the first assessment of hormonal status was performed at 4 months of age, when the infants were still fed only the study formulae, without diversification.

In the present study, the serum IGF-1 concentrations in the breast-fed group was in agreement with the previously reported evolution during the first few months of life in healthy infants fed human milk^(^ ^35^ ^)^. However, differences in the IGF-1 concentration between formula-fed and breast-fed infants were observed. The reason for the difference in IGF-1 concentrations between formula-fed and breast-fed infants is not clear. As we also observed significantly lower serum postprandial insulin and C-peptide concentrations in the breast-fed group, it can be speculated that the difference in IGF-1 concentrations could be related to the higher dietary protein intake in formula-fed infants^(^ ^25^ ^)^. However, a previous randomised controlled study showed no difference in IGF-1 concentrations at 12 months in infants fed cows’ milk compared with those fed formula from 9 to 12 months of age, although the cows’ milk contained twice as much protein as the formula^(^ ^27^ ^)^. This suggests that other factors could influence IGF-1 secretion. In our study, the difference in IGF-1 concentrations between the formula-fed and breast-fed groups lasted longer than the exclusive feeding period, as was previously shown by Madsen *et al*.^(^ ^30^ ^)^. The relationship between breast-feeding and IGF-1 concentrations has been shown to reverse in childhood, as children who had been exclusively breast-fed for at least 2 months during infancy had significantly higher IGF-1 concentrations compared with those who had never been breast-fed in previous studies^(^ ^32^ ^,^ ^36^ ^)^, and it has been reported that these changes in IGF-1 could have long-term effects on adult health^(^ ^37^ ^)^. Among other factors that could influence serum IGF-1 concentrations, it has been suggested that the IGF-1 present in human milk could play a role^(^ ^38^ ^)^. However, there are only very small amounts of IGF-1 present in human milk, and its intestinal absorption is very limited^(^ ^39^ ^)^. Accordingly, some authors have suggested that milk-borne IGF-1 may not be essential for normal healthy infants, owing to the endogenous IGF-1 production^(^ ^40^ ^)^.

At 0·5 months and 4 and 9 months of age, the postprandial insulin and C-peptide concentrations were not different between the F1·8 and F2·7 groups in the present study. These concentrations were higher than those observed in breast-fed infants at 4 months but not at 9 months of age. It has been previously reported that urinary C-peptide concentrations and C-peptide:creatinine ratios are higher in formula-fed infants (2·2 g protein/418·4 kJ (100 kcal)) than in breast-fed infants at 3, 4, 5 and 6 months of age^(^ ^41^ ^)^. In contrast to our findings, Closa-Monasterolo *et al*.^(^ ^42^ ^)^ documented a higher plasma C-peptide:creatinine ratio in the high-protein *v*. low-protein group (no data were presented for the breast-fed group) in the CHOP cohort, although it should be noted that blood samples were taken after 2 months of follow-on formula feeding. Therefore, the markedly higher protein content of the follow-on formula (4·4 *v*. 2·7 g/418·4 kJ (100 kcal) in our study) could explain this difference, as previously mentioned.

In the present study, infants with a higher protein intake exhibited a trend for higher growth parameters up to 60 months of age, with significant differences at 6 and 36 months for body weight and between 6 and 36 months for length. These results are consistent with the body weight and length data presented in the CHOP study^(^ ^10^ ^,^ ^43^ ^)^. In the CHOP study, the body weight-for-age *Z*-scores were significantly higher in the high-protein group at 3, 6 and 12 months but not at 24 months and 6 years of age^(^ ^42^ ^)^. Contrary to our results, the authors reported no differences in the length-for-age *Z*-scores between the high- and low-protein groups at any time point^(^ ^10^ ^,^ ^43^ ^)^. The lack of effect of higher protein intake on the infants fed the high-protein follow-on formula (4·4 g/418·4 kJ (100 kcal), close to cows’ milk protein content) on length in the CHOP study^(^ ^10^ ^,^ ^43^ ^)^ could have been related to the fact that their particularly high protein intake may have surpassed the protein needs for optimal linear growth. Furthermore, kidney size was significantly increased at 6 months of age in the infants fed the high-protein follow-on formula, suggesting an increased, compensatory kidney growth to excrete a larger renal molar load^(^ ^44^ ^)^. Therefore, the data from both the CHOP and EPOCH studies do not support the use of high-protein content in formulae – that is, protein content higher than that in the standard-protein formula used here – during the first few months of life and in follow-on formulae.

As expected, formula feeding and breast-feeding had different effects on growth. During the first 4-month period, both groups of formula-fed infants grew faster than breast-fed infants. However, between 4 and 12 months, breast-fed infants had a higher growth rate, which led to similar growth parameters in formula-fed and breast-fed infants after 12 months. This is in line with the reported lower growth rate of children fed a lower protein diet in the first few months of life^(^ ^12^ ^,^ ^32^ ^)^. As breast-fed infants received a low-protein formula (F1·8) at weaning, their faster growth rate after 4 months of age may have been due to complementary feeding, which provides higher protein intakes compared with exclusively formula-fed infants^(^ ^45^ ^)^. This faster growth rate of breast-fed infants after 4 months persisted up to 60 months of age. At that time, the anthropometric data were similar in the three feeding groups, except for mean head circumference in the F1·8 group, which was significantly lower than that in the F2·7 group at 2 months of life; however, it was similar to that of breast-fed infants, except at 48 and 60 months of age. It is very unlikely that the difference in head circumference could be related to a difference in subcutaneous fat or bone thickness, as there were no differences in BMI, fat mass measured by DEXA or skinfold thickness or in terms of linear growth and bone mass. We were not able to compare these findings with the CHOP study, as head circumference data were not reported^(^ ^10^ ^,^ ^43^ ^)^. Nevertheless, it is noteworthy that the head circumference values in the F1·8 group were within the normal values of the WHO head circumference curves^(^ ^23^ ^)^. Moreover, these results are consistent with those obtained from a Swedish population study showing that infants fed a low-protein formula (1·9 g/418·4 kJ (100 kcal)) tended to have lower head circumference growth during the 1st year of life than breast-fed infants, although the difference was not statistically significant^(^ ^46^ ^)^. Furthermore, the head circumference values in the F1·8 group were slightly greater than the WHO standards, probably because of the differences in the population characteristics compared with the WHO reference population, as was also documented in recently published Scandinavian data^(^ ^47^ ^)^. As the protein content of breast milk at the early stage of lactation is high (2 g/dl)^(^ ^48^ ^)^, which is greater than that in the F1·8 formula (1·8 g/418·4 kJ (100 kcal), i.e. 1·2 g/dl), the protein intake in the breast-fed group may have been significantly higher than that in the F1·8 group during the first few months of life, which could have had a long-term programming effect on growth. Therefore, in future studies evaluating low-protein formulae, it may be necessary to mimic breast milk composition and include a higher protein content (2 g/dl) during the first few weeks of life.

In addition, our data indicate a tendency for higher fat mass in formula-fed infants than in breast-fed infants, although this was statistically significant only during the first 4 months. This is in accordance with the results of Butte *et al*.^(^ ^49^ ^)^ in their study on energy deposition. Fat deposition is influenced by the protein:energy ratio of the ingested milk. As the protein content in human milk is high during the first few weeks of life, its protein:energy ratio is higher, explaining the significantly lower fat mass and higher fat-free mass observed at 0·5 and 4 months of age in the breast-fed group than in the F1·8 group. However, fat mass was similar in the two groups of formula-fed infants. In the CHOP study, fat mass was indirectly measured using isotope dilution in a subgroup of forty-one formula-fed infants at 6 months of age; there were no differences in fat mass, although protein intake was associated with an increase in BMI^(^ ^50^ ^)^. In the present study, when we sequentially measured fat mass by PEA POD and DEXA during the first 60 months of life (Table 6), we found no differences in fat mass between infants fed a low-protein or standard-protein formula. In agreement with our fat mass results, we did not find any differences in the BMI values between infants fed a low-protein or standard-protein formula. These results are different from those of the CHOP study, which reported small, but statistically significant, differences in BMI at 6 years of age between infants fed a high-protein or low-protein formula. These differences were mainly observed in the upper tails of the BMI distribution (95th percentile)^(^ ^43^ ^)^.

The main strength of our study is that we have compared and followed-up prospectively two groups of formula-fed infants in a randomised, double-blinded study with anthropometric data and blood collections and analysis performed at one site, with strict standardised protocols. In addition, very few infants were lost to follow-up.

Acknowledging the fact that this is a single-centre study, there are some potential limitations to our study. The main limitation in interpreting the statistical significance of data after 4 months of age is the number of patients included. This number was calculated for the primary outcome, and our data show that the IGF-1 blood levels of infants fed formula with low protein (F1·8) or high protein (F2·7) content did not differ at 4 and 9 months of life. The absence of a difference in IGF-1 concentrations at 4 months is not likely to be related to a lack of power, as the standard deviation of IGF-1 measured in our study was lower than the value used for sample size calculation, and the number of subjects analysed was higher than that required, due to the low rate of patient loss to follow-up during the study. As the number of infants was not calculated to show statistical differences in growth parameters, we have to be cautious in drawing conclusions in terms of growth. However, as differences in the head circumference growth pattern between the two study formulae were observed, beginning at 2 months and at each following anthropometric measurement, we believe that these differences would have persisted with a greater number of infants. Moreover, it is possible that the adjustment of lactose content to keep the same energy density in the two formulae could have had an effect on the serum IGF-1 concentrations. However, is very unlikely that such an increase in lactose content in F1·8 could have an effect on serum IGF-1 concentrations, as we did not observe any difference in glycaemia or in insulin and C-peptide levels between the two groups of formula-fed infants. Another limitation in comparing data with recent publications could be the fact that the follow-on formula fed to our infants was the same as the initial formula. This could explain the discrepancies observed between our results and data from other studies that changed the follow-on formula to one with a much higher protein intake (close to cows’ milk) after the 4th month of life^(^ ^10^ ^,^ ^37^ ^)^.

In conclusion, within the range of protein content of the formulae studied, we were not able to observe a difference in IGF-1 serum levels at 4 months of life. At 60 months of age, infants fed these formulae exclusively for 4 months and as a follow-on formula up to 1 year exhibited no differences in growth, with the exception of head circumference, which was lower in infants fed the low-protein formula compared with infants fed the standard-protein formula. Nevertheless, the mean head circumference value remained above the WHO growth standard.

## Supplementary material

For supplementary material accompanying this paper visit http://dx.doi.org/10.1017/S0007114515004456. click here to view supplementary material
